# Comparative genomic analysis of *Bacillus paralicheniformis* MDJK30 with its closely related species reveals an evolutionary relationship between *B. paralicheniformis* and *B. licheniformis*

**DOI:** 10.1186/s12864-019-5646-9

**Published:** 2019-04-11

**Authors:** Yuhui Du, Jinjin Ma, Zhiqiu Yin, Kai Liu, Gan Yao, Wenfeng Xu, Lingchao Fan, Binghai Du, Yanqin Ding, Chengqiang Wang

**Affiliations:** 1 College of Life Sciences / National Engineering Laboratory for Efficient Utilization of Soil and Fertilizer Resources / Shandong Key Laboratory of Agricultural Microbiology, Shandong Agricultural University, Tai’an, People’s Republic of China; 2 Key Laboratory of Molecular Microbiology and Technology of the Ministry of Education, TEDA College, Nankai University, Tianjin, People’s Republic of China; 3 State Key Laboratory of Nutrition Resources Integrated Utilization, Linshu, People’s Republic of China

**Keywords:** PGPR, *Bacillus paralicheniformis*, *Bacillus licheniformis*, Comparative genomics, Secondary metabolites

## Abstract

**Background:**

Members of the genus *Bacillus* are important plant growth-promoting rhizobacteria that serve as biocontrol agents. *Bacillus paralicheniformis* MDJK30 is a PGPR isolated from the peony rhizosphere and can suppress plant-pathogenic bacteria and fungi. To further uncover the genetic mechanism of the plant growth-promoting traits of MDJK30 and its closely related strains, we used comparative genomics to provide insights into the genetic diversity and evolutionary relationship between *B. paralicheniformis* and *B. licheniformis.*

**Results:**

A comparative genomics analysis based on *B. paralicheniformis* MDJK30 and 55 other previously reported *Bacillus* strains was performed. The evolutionary position of MDJK30 and the evolutionary relationship between *B. paralicheniformis* and *B. licheniformis* were evaluated by studying the phylogeny of the core genomes, a population structure analysis and ANI results. Comparative genomic analysis revealed various features of *B. paralicheniformis* that contribute to its commensal lifestyle in the rhizosphere, including an opening pan genome, a diversity of transport and the metabolism of the carbohydrates and amino acids. There are notable differences in the numbers and locations of the insertion sequences, prophages, genomic islands and secondary metabolic synthase operons between *B. paralicheniformis* and *B. licheniformis*. In particular, we found most gene clusters of Fengycin, Bacitracin and Lantipeptide were only present in *B. paralicheniformis* and were obtained by horizontal gene transfer (HGT), and these clusters may be used as genetic markers for distinguishing *B. paralicheniformis* and *B. licheniformis*.

**Conclusions:**

This study reveals that MDJK30 and the other strains of lineage *paralicheniformis* present plant growth-promoting traits at the genetic level and can be developed and commercially formulated in agriculture as PGPR. Core genome phylogenies and population structure analysis has proven to be a powerful tool for differentiating *B. paralicheniformis* and *B. licheniformis*. Comparative genomic analyses illustrate the genetic differences between the *paralicheniformis-licheniformis* group with respect to rhizosphere adaptation.

**Electronic supplementary material:**

The online version of this article (10.1186/s12864-019-5646-9) contains supplementary material, which is available to authorized users.

## Background

Plant growth-promoting rhizobacteria (PGPR) are a group of rhizosphere bacteria that promote plant growth [1]. PGPR competitively colonize the plant rhizosphere and can simultaneously act as biofertilizers and as competitors of plant-pathogenic bacteria and fungi [2]. The genus *Bacillus* comprise typical species of PGPR that can suppress some plant pathogens by producing antibiotics, including Bacilysin [3], Difficidin [4], Iturin A [5], and Surfactin [6]. Some *Bacillus* strains can be used as soil inoculants in agriculture and horticulture for plant growth promotion and biocontrol. Bacterial secondary metabolism is a rich source of novel bioactive compounds with potential pharmaceutical applications as antibiotics [7]. Antibiotic production by *Bacillus* spp. enhances the fitness of the production strains and suppresses plant pathogens that would otherwise harm plant health. These antibiotics mainly belong to nonribosomal peptides and polyketides, which are synthesized by nonribosomal peptide synthetases (NRPS) and polyketide synthases (PKS), respectively [8]. NRPS and PKS are modular and composed of a series of domains including adenylation, thiolation, condensation and esterification domains [9].

*B. paralicheniformis* is a gram-positive, facultative anaerobic, motile *Bacillus* species [10]. It has been reported that *B. paralicheniformis* is most closely related to *B. licheniformis* and *B. sonorensis*, based on phylogenetic analysis [10]. *B. paralicheniformis* and *B. licheniformis* have been used for decades in the biotechnology industry to manufacture enzymes, antibiotics, biochemicals and consumer products [10, 11]. Some strains of these species are used to produce peptide antibiotics such as Bacitracin, Fengycin, Lichenysin and Lantipeptide [12, 13]. Thus, some isolates within the *paralicheniformis-licheniformis* group can mitigate the effects of fungal pathogens on field crops [14, 15]. Dunlap et al. have distinguished between *B. paralicheniformis* and *B. licheniformis* based on phylogenetic and phenotypic analyses. However, phylogenetic and genetic characteristics may be ambiguous within the *paralicheniformis-licheniformis* group.

*B. paralicheniformis* MDJK30 was recently isolated and identified from the rhizosphere of peony in Shandong, China. Its complete genome sequence has been reported [14]. To reveal the evolutionary relationship between *B. licheniformis* and *B. paralicheniformis*, in this study, MDJK30 together with 55 other previously published *Bacillus* strains was comparatively studied by phylogenetic analysis, population genetic structure analysis and ANI results. Functional and comparative genomics analysis provided a better understanding of genome evolution and hereditary differences within the *paralicheniformis-licheniformis* group. Our results revealed genomic differences in secondary metabolic gene clusters between *B. paralicheniformis* and *B. licheniformis*, suggesting their roles in adaptation to various antibiotic stressors in the rhizosphere.

## Results

### General features of *B. paralicheniformis* MDJK30 as PGPR

**Fig. 1:**
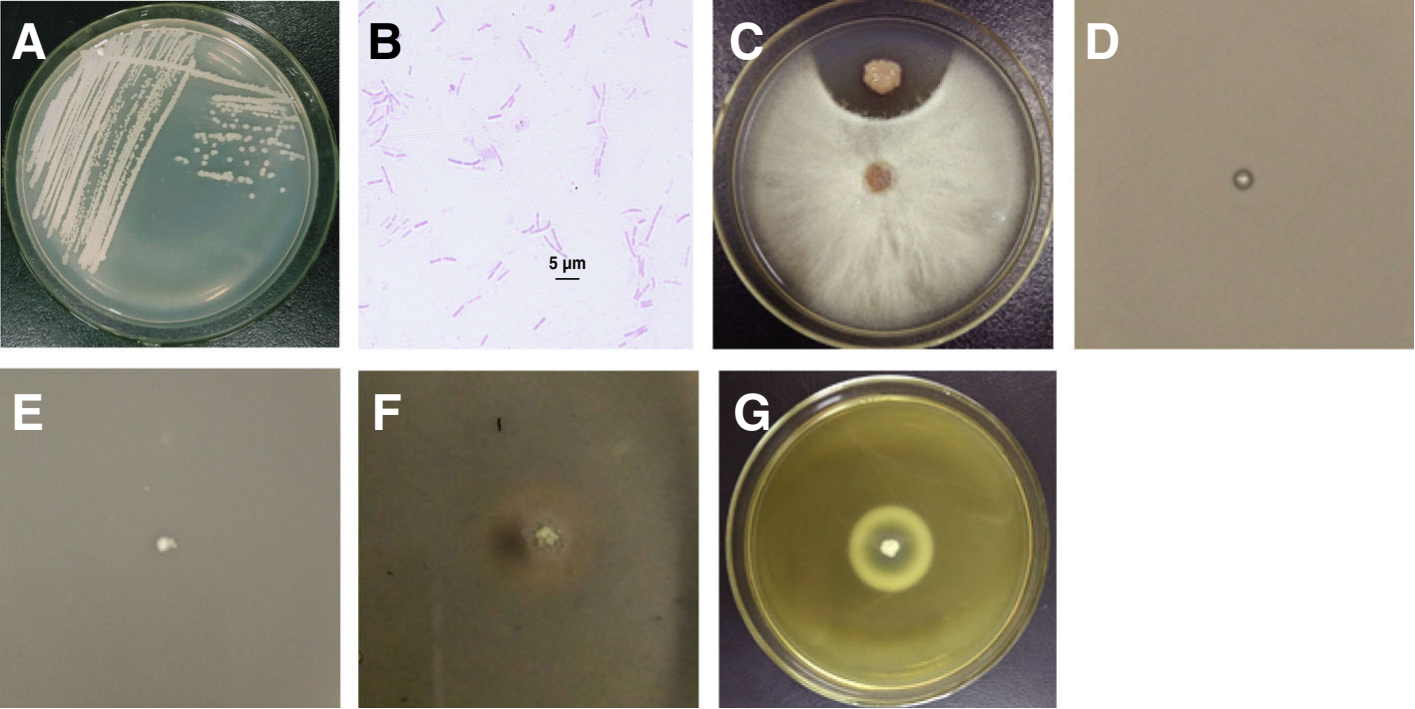
Morphological characteristics and antagonistic activities of B. paralicheniformis MDJK30. (**a**) Cellular morphology (magnification 10 × 100). (**b**) MDJK30 was inoculated on LB agar medium and incubated at 37 °C for 24 h. Strains were stained with 0.5% magenta dye. In vitro antagonistic activities of *B. paralicheniformis* MDJK30 against *F. solani* (**c**), *B. subtilis* (**d**), and *E. coli* (**e**). The antifungal activity of MDJK30 was determined against *F. solani*. A newly cultivated hyphal plug of *F. solani* was placed on the center of a new PDA plate and incubated for 1 day at 28 °C, and a single clone of MDJK30 was inoculated onto one side of the plug at a distance of 2 cm to incubate for another 3 days. The antibacterial assays for MDJK30 were performed against *E. coli* and *B. subtilis*. The precultured *E. coli* or *B. subtilis* were incubated in 5 mL LB liquid medium for 10 h at 37 °C. Then, 1 mL of the culture was mixed with 20 mL LB semisolid medium. A single colony of MDJK30 was placed on the center of cooled medium to incubate for 1 day at 37 °C. Qualitative analysis of siderophores (**f**) A single clone of *B. paralicheniformis* MDJK30 was inoculated on a CAS-agar plate for cultivation for 3 days at 37 °C. The presence of an orange ring around the colony suggested the capacity for producing siderophores to chelate iron in the medium. Analysis of casein degradation (**g**) A single clone of *B. paralicheniformis* MDJK30 was inoculated in the center of casein medium and cultivated for 2 days at 37 °C

*B. paralicheniformis* MDJK30 is a rod-shaped, gram-positive bacterium that produces endospores. When cultivated on LB, it forms a beige, dry, and irregular colony (Fig. 1 a and b). To test the antagonistic activity of MDJK30 to fungi and bacteria, we performed the dual culture assay test against *Fusarium solani*, *B. subtilis* 168, and *Escherichia coli* DH5α, respectively. The results (Fig. 1c, d and e) showed that MDJK30 had high antagonistic activity to *F. solani* and *B. subtilis* but no effect on *E. coli*, which indicated the potential application for controlling pathogenic fungi and gram-positive bacteria. As a member of PGPR, MDJK30 can produce siderophores (Fig. 1f) and shows casein degradation activity (Fig. 1g). Due to the multiple beneficial effects of MDJK30, this strain can be developed and commercially formulated, either alone or as part of a microbial consortia, for field application to control plant pathogens and promote crop growth.

**Fig. 2:**
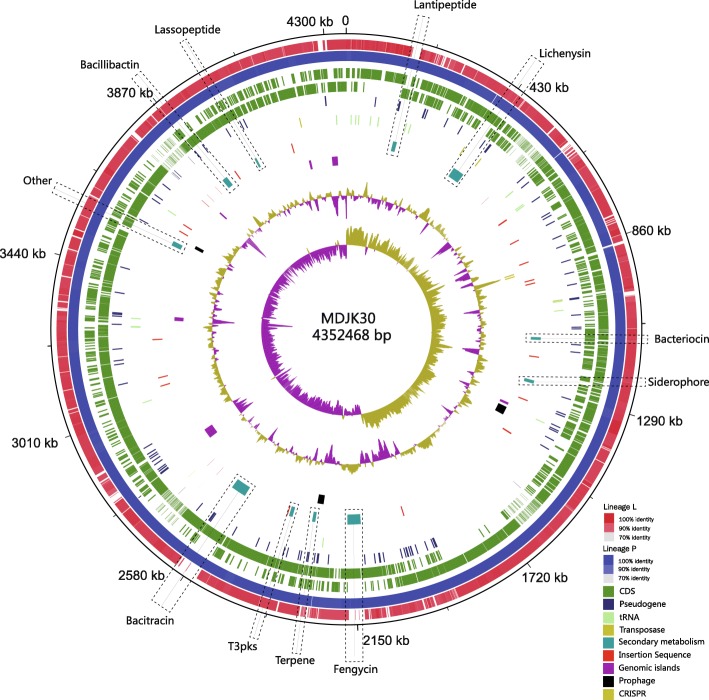
Circular Representation of *B. paralicheniformis* MDJK30. Rings represent the following features labelled from outside to center, where the outermost circle represents the scale in bps. 1st ring; combined genomic regions of lineage L strains (red), 2nd ring; combined genomic regions of lineage P strains (blue), 3rd ring; plus-strand CDS (navy blue), 4nd ring; minus-stand CDS (navy blue), 5rd ring; pseudogene (light green), 6th ring; secondary metabolism gene clusters (blue green), Insertion sequence (red), 7th ring; genomic islands (purple), prophage (black), CRISPR (yellow), 8th ring; GC-plot, yellow and purple correspond to above- and below-average GC content respectively, 9th ring; GC-skew, yellow and purple correspond to above- and below-average GC skew, respectively

To further uncover the molecular mechanisms of plant growth promotion, the complete genome sequence of MDJK30 was sequenced and deposited at GenBank (accession number CP020352) [14] (Fig. 2). The MDJK30 chromosome is interrupted by numerous mobile elements, including 2 CRISPRs, 5 transposases, 3 prophage/prophage-like elements, 23 insertion sequence, and 6 genomics islands (Additional file 1:Table S1). The existence of mobile genetic elements is indicative of HGT.

### Secondary metabolism gene clusters in MDJK30

MDJK30 has the potential to synthesize bioactive secondary metabolites. Our comparative genomic analysis of MDJK30 identified 11 gene clusters (Fig. 2) related to secondary metabolites using antiSMASH [16]. There were four gene clusters (Lichenysin, Fengycin, Bacitracin, Bacillibactin) belonging to non-ribosomal peptide synthesis. The representative gene clusters encoding NRPS and the putative product structure are summarized in Additional file 2: Figure S1A. Lichenysin is a hemolytic surfactin-like toxin with activity against gram-positive and gram-negative bacteria [17]. Fengycin, as a cyclic lipopeptide, is reportedly to have strong antifungal activity, specifically against filamentous fungi [13]. The iron-siderophore Bacillibactin may enhance the ability of MDJK30 to scavenge iron from the rhizosphere [18]. The other seven secondary metabolism gene clusters include Lantipeptide, Bacteriocin, Siderophore, Terpene, Lassopepetide, T3pks and Other (unknown). These biosynthetic gene clusters are represented in Additional file 2: Figure S1B. Furthermore, we compare the homology of these gene clusters. The homologous gene clusters of MDJK30 are represented in Additional file 3: Figure S2. Notably, some gene clusters in MDJK30 show high homology to those from other species, such as *B. halodurans, B. subtilis* and *Pontibacillus litoralis*. These data suggest that the secondary metabolism gene clusters were horizontally transferred among *B. paralicheniformis* and other species.

### Phylogenetic analyses

**Fig. 3:**
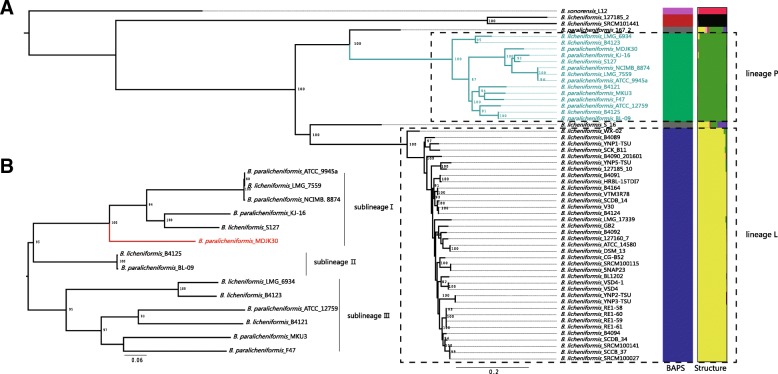
Phylogenetic analysis and population structure of 56 *Bacillus* strains. (**a**) Maximum likelihood tree was constructed using PhyML based on 528 single-copy core genes shared by 56 *Bacillus* strains. The 14 lineage P strains are indicated in mint green. Next to the tree, colored blocks indicate the BAPS and STRUCTURE clusters. (**b**) Maximum likelihood tree of 14 lineage P strains. *Bacillus paralicheniform*is MDJK30 is indicated in red. The interior node values of the tree are bootstrap values (100 replicates), values higher than 80 are indicated

It has been reported that *B. paralicheniformis* is most closely related to *B. licheniformis* and *B. sonorensis*. Our collection included 9 *B. paralicheniformis* strains, 46 *B. licheniformis* strains and 1 *B. sonorensis* strains. The main features of the *B. paralicheniformis* MDJK30 genome and other strains in the current study are summarized in Additional file 4: Table S2. To assess the phylogenetic position of MDJK30 and the phylogeny of *B. licheniformis* and *B. paralicheniformis*, we constructed a maximum likelihood phylogenetic tree based on the alignment of nucleotide sequences for the 528 single-copy genes (Additional file 5: Table S3) shared by all strains in the current research using PhyML [19], for which one *B. sonorensis* strain was designed as an outgroup (Fig. 3a). To further explore the genomic similarities among strains, we constructed population genetic structure analysis using Bayesian Analysis of Population Structure (BAPS) [20] and the program STRUCTURE [21]. Population structure analysis assigned the *B. licheniformis* and *B. paralicheniformis* complex to multiple clusters (Fig. 3a), which generally correspond to well-resolved clades or subclades in the phylogenetic tree. The phylogenetic tree showed that most strains fall predominantly into two distinct phylogenetic lineages designated lineages L (lineage *licheniformis*) and P (lineage *paralicheniformis*). Lineage P and L form distinct, extremely tight clusters on separate clades from other strains. A small number of strains are distributed outside the lineage L and P. Two strains (127185_2 and SRCM101441) fall into a monophyletic clade, and the other two strains (167_2 and S_16) are singletons. Furthermore, the ANI results validated the conclusion of the phylogenetic analysis. As shown in Additional file 6: Table S5, all strains of lineage L and P resulted in a higher ANI (lineage L > 99.13% and lineage *P* > 98.6%), which was much higher than the cut-off value of 95% for distinguishing different species [22]. The genomes of the lineage P strains were slightly larger than (4.29 ± 0.123 vs 4.22 ± 0.197 Mbp) and had similar GC contents (46.81 ± 0.31 vs 46.85 ± 0.12%) to the genomes of the lineage L strains.

Lineage L contains 37 strains identified as the species *licheniformis*. MDJK30 and the other 13 strains form a monophyletic lineage P and evolve from a common ancestor. Meanwhile, 6 strains of lineage P were previously identified as species *licheniformis*. Our phylogenetic analysis and ANI results indicated that there are mistakes in the classification of strain B4121, B4125, B4123, LMG 6934, LMG 7559 and S127, which should be corrected to species *paralicheniformis*. This mis-classification was also reported in other strains (*B. paralicheniformis* ATCC 9945a were initially identified as *B. licheniformis*) and have generally been corrected with advances in technology [22–24].

A total of 1718 single-copy core genes (Additional file 7: Table S4) were identified by comparison of the 14 lineage P genomes. The strains of lineage P have similar genetic characteristics, with more core genes and similar genome sizes (3.9–4.5 Mb), and the numbers of CDSs range from 4026 to 4859. We constructed a maximum likelihood tree based on the alignment of nucleotide sequences for 1718 single-copy genes shared by all lineage P strains. Furthermore, we also constructed a Neighbor-joining tree using MEGA for 56 *Bacillus* strains and all lineage P strains, respectively (Additional file 8: Figure S3). The core genome phylogeny of lineage P illustrates the diversity of *B. paralicheniformis*. The phylogenetic tree of lineage P was divided into three distinct sublineages with high bootstrap support values (Fig. 3b). MDJK30 and five other strains (NCIMB_8874, LMG_7557, ATCC_9954a, KJ-16, and S127) form a sublineage I suggest that MDJK30 is closely related to these five strains. Sublineage II contains two strains (B4125 and BL-09), and sublineage III contains 6 strains (ATCC_12759, B4121, LMG_6934, B4123, MKU3, and F47).

### Comparative genomic analysis

**Fig. 4:**
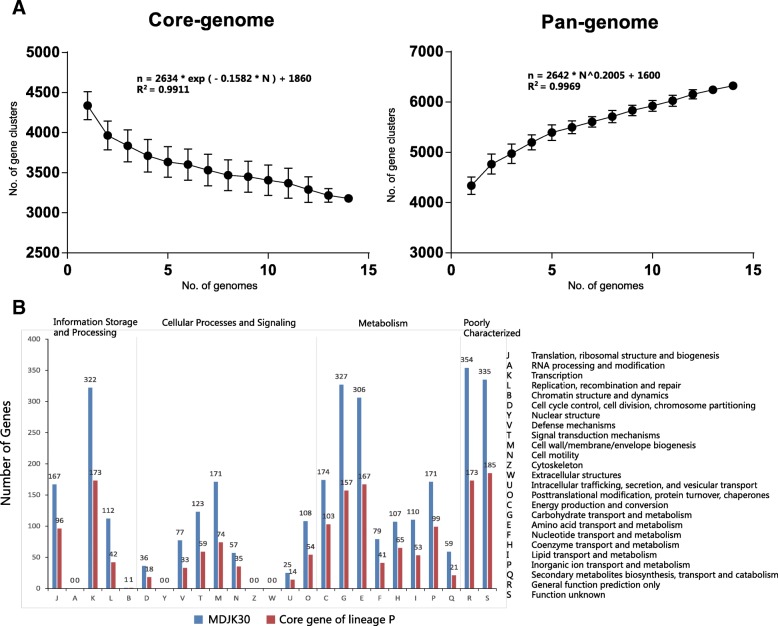
Genomic characteristics and COG annotation of MDJK30 and core genome of lineage P. (**a**) Core- and pan genome graphs of lineage P. The two curve shows that the downward trend of the core gene clusters and the upward trend of the pan gene clusters with the increasing number of genomes, respectively. Black spots are the averages of such values. Error bars indicate variations in the number of the core and pan-gene clusters among different strains. The deduced mathematical function is also reported. (**b**) COG functional categorization of MDJK30 and core genome of lineage P

To assess genetic diversity, we identified the core and pan genomes across all lineage P strains. A pan genome of 6106 genes and core genome of 2068 genes were observed in lineage P. The pan genome content of lineage P, when plotted on a log-log scale versus the number of genomes, shows a clear linear upward trend in coincidence with the Heap’s power law pan-genome model [25] with positive exponent y = 0.2005 (Fig. 4a). The exponent y > 0 indicates an open genomes species [25]. 16.6% (1012 out of 6106) of the genes in the pan genome were present in only one genome of lineage P, suggesting the occurrence of a large number of horizontal transfer events. In contrast to the pan genome, the number of core genomes shared across species decreased sharply, reaching a minimum value of 2068 for all lineage P strains analyzed (Fig. 4a). We also used a mathematical model to estimate the minimum number of core genes by fitting a single exponential decay function. The predicted minimum core gene content of lineage P was 1860.

We further used Cluster of Orthologous Group (COG) assignment [26] to determine into which functional category the genes of MDJK30 and core genome of lineage P fall. The number of genes assigned to each COG is present in Fig. 4b. As show in COG assignment, a higher proportion of the genes of MDJK30 were assigned to the K (9.8%, transcription), G (10.0%, carbohydrate transport and metabolism) and E (9.3%, Amino acid transport and metabolism) categories. The core genome of lineage P has a consistent functional category by COG compared with MDJK30, and as indicated, despite their geographical isolation and varied associated-plant, the majority of genes implicated in rhizosphere adaptation and competitiveness were highly conserved among the *B. paralicheniformis* strains.

**Fig. 5:**
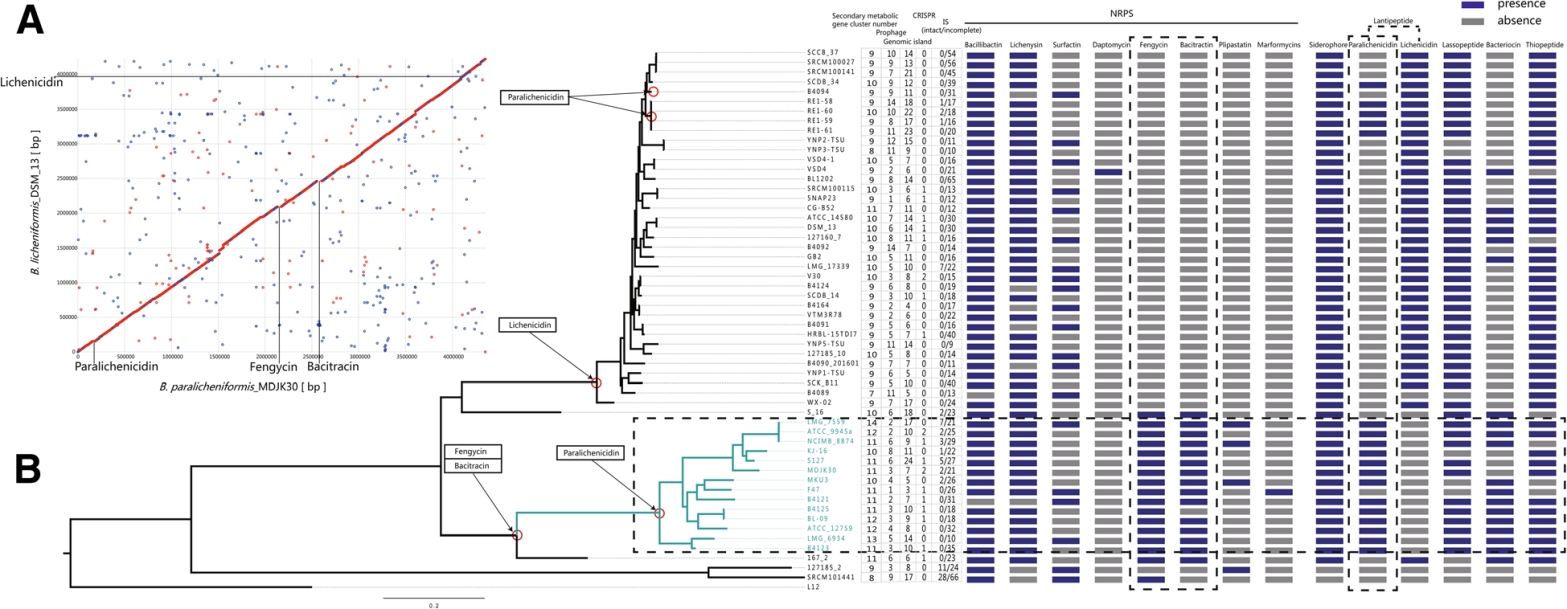
The secondary metabolic gene clusters evolution. (**a**) MUMmer plot of *B. paralicheniformis* MDJK30 against *B. licheniformis* DSM 13 based on nucleotide sequences. (**b**) Number and distribution heatmap of secondary metabolic gene clusters identified among lineage P strains and lineage L strains. Color coding for genes is based on the Blast score ratio (BSR) recorded for each genome when screened with genes of MDJK30. Arrows and red circle indicate estimated points of insertion of independently acquired secondary metabolism gene cluster

Comparative genomic and phylogenetic analysis within the *paralicheniformis-licheniformis* group may provide new information regarding the evolution, ecology and differences between these two closely related species. The shared regions between the genomes of *B. paralicheniformis* MDJK30 and the reference strain *B. licheniformis* DSM 13 show 94.7% nucleotide identity and broad organizational similarity (Fig. 5a). Despite the extensive colinearity of the *B. paralicheniformis* and *B. licheniformis* genome, there are some *B. paralicheniformis*-specific genome segments, including insertion sequence, prophage-like elements, and secondary metabolism synthases that are not present in *B. licheniformis* (Fig. 2). It is indicated that the presence of these genes promotes the adaptation of *B. paralicheniformis* to grow in additional environmental niches compared to *B. licheniformis*. In general, the phylogenetic relationship and genomic differences may imply overlapping, but species-specific environmental niches.

The *paralicheniformis-licheniformis* group includes many strains that are widely used in biotechnology industry and field applications. Their genomes harbor many gene clusters related to secondary metabolic synthases. The availability of many genomes from the *paralicheniformis-licheniformis* group should permit a thorough comparison of these secondary metabolic synthases in *B. paralicheniformis* and *B. licheniformis*, thereby offering new perspectives and strategies for facilitating future applications in agriculture and industry. Genome mining identified a large number of gene clusters related to secondary metabolic synthases in the *paralicheniformis-licheniformis* group. To further elucidate the evolution and distribution of these gene clusters, we located and screened these gene clusters in the pan genome and identified the genomic events contributing to the differentiation of rhizosphere adaptation between *B. paralicheniformis* and *B. licheniformis* during the evolutionary process. The distribution heatmap and possible evolutionary node are represented in Fig. 5b. As shown in Fig. 5b, lineage L, lineage P and lineage 2 have common, identical biosynthesis gene clusters (Lichenysin, Bacillibactin, Bacteriocin, Siderophore, Lassopeptide, part of Terpene and Fengycin), in agreement with previous studies [10, 27]. In some secondary metabolism gene clusters, additional features such as adjacent tRNA, prophage, transposases, insertion sequence and CRISPRs are indicative of HGT (Fig. 2). Our analysis shows that most strains of lineage P contain consistent biosynthetic genes, suggesting that despite their geographical isolation and varied associated plants, the majority gene clusters implicated in rhizosphere adaptation and competitiveness were highly conserved among *B. paralicheniformis* strains. In addition, some strains do not contain certain gene clusters, due to a simple deletion event.

Interestingly, two non-ribosomal peptide and one specific Lantipeptide synthase genes found in lineage P but absent in lineage L are those involved in the synthesis of Bacitracin, Fengycin and paralichenicidin (Fig. 5b). The deviant GC content is used as a detection method for HGT [28]. We detected the GC content of secondary metabolism gene clusters, which displayed an apparent deviation with their host genomes (Additional file 9: Figure S4). Five strains of lineage L also have Lantipeptides, suggesting that two additional HGT events occurred within lineage L. These gene clusters (Fengycin, Bacitracin and Lantipeptide) were performed to explore the differences in the categorical genome and rhizosphere adaptation between *B. paralicheniformis* and *B. licheniformis*.

### Specific secondary metabolism gene clusters in *B. paralicheniformis*

#### Fengycin

**Fig. 6:**
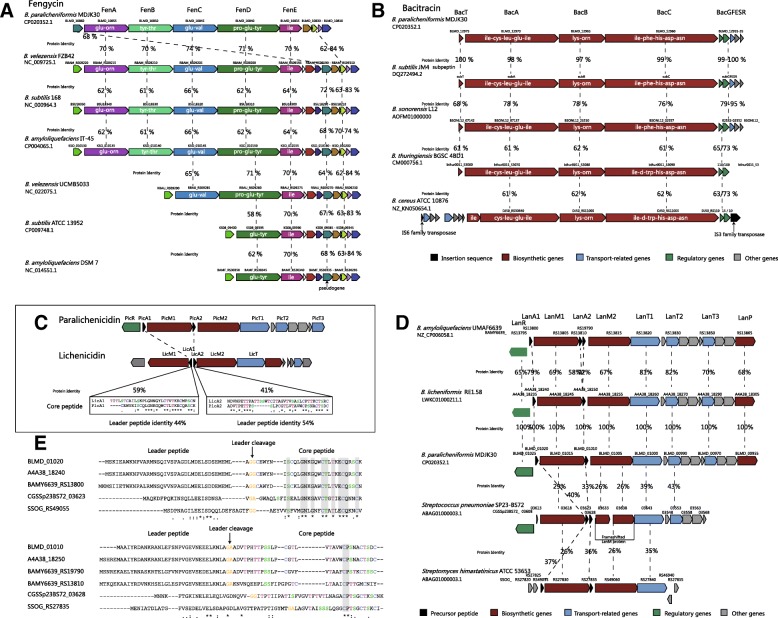
Genome mining of Fengycin, Bacitracin and Paralichenicidin-like gene clusters. (**a**) Genetic organization of gene clusters of Fengycin. Same genes are shown in the same color and linked by dotted lines. (**b**) Genetic organization of gene clusters of Bacitracin. Percentage amino-acid identities of each peptide with Bacitracin operon in *B. paralicheniformis* MDJK30 are shown. Insertion sequence, biosynthetic genes, transport genes, regulatory genes and other genes are shown in black, red, blue, green and gray, respectively. A domain substrates in biosynthetic genes are predicted by antiSMASH [16]. (**c**) Genetic organization of gene clusters of Paralichenicidin and Lichenicidin. Region in inset box compares two amino acid sequences of the core peptide LanA1 and LanA2 of the structural components of Paralichenicidin and Lichenicidin. (**d**) Genetic organization of related gene clusters of Paralichenicidin. Percentage amino acid identities of each peptide with Paralichenicidin peptide are shown. (**e**) Sequence alignment of LanA1 and LanA2. Completely conserved and highly conserved residues are indicated by asterisks and points, respectively. Completely conserved residues in core peptides are shown in gray boxes. Ser, Thr and Cys residues in the core peptide are shown in purple, green and blue, respectively. Leader cleavage site is predicted by antiSMASH [16] and shown in yellow

**Fig. 7:**
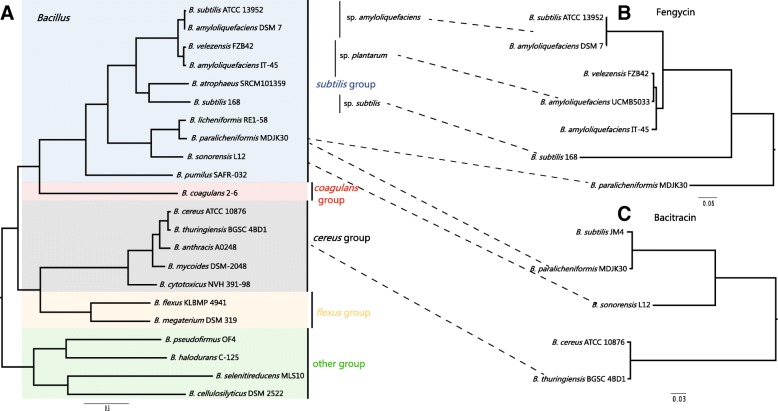
Maximum-likelihood (ML) phylogeny of Species, Fengycin and Bacitracin. (**a**) ML species phylogeny. The *Bacillus* tree set into groups containing phylogenetically related species: the *subtilis*, *coagulans*, *cereus*, *flexus* and “other” groups reported previously [66]. (**b**) ML phylogeny of six protein sequences in Fengycin gene clusters (BLMD_10810/ 10,815/ 10,820/ 10,830/ 10,835/ 10,840). (**c**) ML phylogeny of five protein sequences in Bacitracin gene clusters (BLMD_12935/ 12,940/ 12,960/ 12,965/ 12,970)

Fengycin (synonymous to Plipastain) that specifically acts against filamentous fungi is biosynthesized by Fengycin synthetase consisting of the five NRPSs FenA-FenE encoded by *fenA-E* [13, 29]*.* Fengycin has been isolated from members of the *Bacillus* genus [30]. The closely related gene cluster for Fengycin biosynthesis of *B. paralicheniformis, B. amyloliquefaciens, B. subtilis* and *B. velezensis* inhabits identical gene loci (Fig. 6a), suggesting that they have evolved from a common ancestor and/or might be interchangeable genetic elements. Additionally, the phylogenetic analysis revealed a narrow cluster distribution (*subtilis* group), and the consistent phylogeny between Fengycin cluster tree and species tree, suggesting that this cluster may have been originated via an HGT event from a donor closely related to the subtilis order (Fig. 7).

Interestingly, there is an incomplete Fengycin cluster found in some strains of *B. subtilis* and *B. amyloliquefaciens*, such as *B. subtilis* ATCC 13952 and *B. amyloliquefaciens* DSM 7. In this incomplete Fengycin cluster, the biosynthetic genes for the *fenA-C* and part of *fenD* modules were absent. The strains ATCC 13952 and DSM 7 are non-plant-associated [31]. Moreover, most strains containing the complete Fengycin cluster are plant-associated strains [31]. Therefore, we hypothesized that gene loss events related to the biosynthetic genes of Fengycin production occurred in non-plant-associated strains. This hypothesis consists with previous studies that Fengycin prevents the growth of a wide range of plant pathogens, especially filamentous fungi [32].

#### Bacitracin

The Bacitracin operon in *B. paralicheniformis* MDJK30 contains multiple genes, namely, Bacitracin synthetase (*bacA*: BLMD_12,970, *bacB*: BLMD_12,965 and *bacC*: BLMD_12,960), thioesterase (*bacT*: BLMD_12,975), those of a two-component regulatory system (*bacR*: BLMD_12955 and *bacS*: BLMD_12950), an ABC transport gene (*bacE*: BLMD_12945) and two other genes (*bacG*: BLMD_12935 and *bacF*: BLMD_12,940), that may not be involved in Bacitracin production. In previous studies, there was observed variation in the prevalence of Bacitracin synthase genes among *B. licheniformis* strains, and approximately 50% may harbor the *bac* operon [11, 33]. More remarkably, the *bac* operon is not present in the reference strain (DSM 13/ATCC 14580) genome [11]. In this study, our comparative genomic analysis clearly illustrates the distribution of the *bac* operon in the *paralicheniformis-licheniformis* group, which indicates that this operon is present only in the strains of lineage P. These genes are also absent from all strains of lineage L. A previous study showed that erythromycin resistance of *paralicheniformis-licheniformis* group isolates is independent of Bacitracin production [34]. This indicates that Bacitracin production and erythromycin resistance could be an important characteristic specific to *B. paralicheniformis*. and *B. subtilis* has been shown to produce a Bacitracin called Subpeptin [35]. The Bacitracin synthetase of lineage P displayed a significant sequence similar to subpeptin with approximately 97–100% identity to the protein sequence (Fig. 6b). Genes for Subpeptin synthesis have been described for *B. subtilis* JM4 but are absent in most *B. subtilis* strains. It is likely that the gene cluster for Subpeptin synthesis was acquired from *B. paralicheniformis* through a recent single event of HGT. Furthermore, we found a putative Bacitracin operon in *B. sonorensis*, *B. thuringiensis* and *B. cereus* using the ClusterBlast service in antiSMASH [16]. The Bacitracin cluster is only found in some species of the *cereus* and *subtilis* groups, and is absent in the *flexus*, *coagulans* and other groups. The phylogeny of this cluster was consistent with the *Bacillus* species tree, as expected under the hypothesis the Bacitracin cluster was transferred from a donor closely related to the ancestor order of *cereus*, *coagulans* and *subtilis* groups via an HGT event (Fig. 7). The Bacitracin synthetase of *B. sonorensis*, *B. thuringiensis* and *B. cereus* revealed 76–78%, 61–62% and 61–62% identity with the Bacitracin operon of *B. paralicheniformis*, respectively (Fig. 6b). Although the synthetase genes of the Bacitracin operons in different strains show a certain degree of divergence in sequence, the substrates of the A domains were conserved. As shown in Fig. 6b, the substrates of the A domains in BacA, BacB and BacC were determined to be ile-cys-leu-glu-ile, lys-orn and ile-phe/d-trp-his-asp-asn, respectively. More remarkably, the amino acids of the second A domain in BacC could be modified phe or d-trp, which produced two types of Bacitracin (ile-cys-leu-glu-ile-lys-orn-ile-phe-his-asp-asn and ile-cys-leu-glu-ile-lys-orn-ile-d-trp-his-asp-asn). This difference might hint at the biological differences in Bacitracin production in different strains. *bacR* and *bacS* are conserved in all Bacitracin operons of different strains, and their products make up the regulator and sensor proteins of the two-component systems. This two-component system plays a key role in the Bacitracin self-resistance of the host strains producing Bacitracin [36, 37]. *bacE, bacF, bacG* and *bacT* are absent in *B. thuringiensis* and *B. cereus*, indicating they might not be important to Bacitracin production. Furthermore, in *B. cereus* ATCC 10876, the Bacitracin operon is located between two types of IS elements, IS6 and IS3. It appears likely that the acquisition of this Bacitracin operon is mediated by its flanking IS elements. In general, these data suggest that the two-component systems (*bacR* and *bacS*) and three synthetase genes (*bacA*, *bacB* and *bacC*) make up the basic interchangeable genetic elements of the Bacitracin operon.

#### Paralichenicidin

Lantipeptide represents a potential antimicrobial strategy against a range of pathogenic bacteria [30]. The Lantipeptide in MDJK30 is a two-peptide Lantipeptide, named Paralichenicidin. The gene cluster of Paralichenicidin is located on both strands at the start of the genome and covers 162,067 to 179,818 bp (BLMD_00955 to BLMD_01025) (Fig. 6d). The GC content (38.6%) of the cluster is quite different from the average GC content of the whole genome (46.7%) (Additional file 9: Figure S4). Analysis of the Paralichenicidin operon identified three transport proteins (BLMD_01000: LanT1, BLMD_00990: LanT2 and BLMD_00970: LanT3), two Lantipeptide structural proteins (BLMD_01010: LanA1 and BLMD_01020: LanA2), three modification enzymes (BLMD_01005: LanM1, BLMD_01015: LanM2 and BLMD_00955: LanP) and one regulator (BLMD_01025: LanR). In comparison to Paralichenicidin and Lichenicidin, the core peptide of PicA1 and PicA2 revealed 41 and 59% identity with the LicA1 and LicA2 of lichenicidin, respectively (Fig. 6c). Interestingly, five strains of lineage L have two Lantipeptide-encoding gene clusters, namely, for Lichenicidin and Paralichenicidin (Fig. 5b). The five Paralichenicidin-encoding gene clusters in lineage L strains may thus be the result of multiple HGT from the *B. paralicheniformis* strains.

To investigate the putative homologs of Paralichenicidin, we performed a genome-wide examination of the LanMs associated with Paralichenicidin synthetase analogues, which involved a PSI-BLAST search of the NCBI non-redundant protein database. All results with significant E values were examined and further manually reviewed according to the gene orders and genes downstream and upstream of the LanM homologs. Of the potential homologs, three were selected for closer inspection, including *B. amyloliquefaciens* UMAF6639 (Amyloliquecidin, BAMY6639_13865 to BAMY6639_RS13795), *Streptococcus pneumoniae* SP23-BS72 (CGSSp23BS72_03568 to CGSSp23BS72_03608), and *Streptomyces himastatinicus* ATCC 53653 (SSOG_RS27855 to SSOG_RS27820) (Fig. 6d).

Amyloliquecidin, a known two-component Lantipeptide produced by *B. amyloliquefaciens*, is a recommended treatment for *Staphylococcus aureus*-induced skin infections [38]. Two propeptides (AmyA1|BAMY6639_RS13800: LanA1 and AmyA2|BAMY6639_RS19790: LanA2) revealed 79 and 72% identity with PicA1 and PicA2, respectively. As with Paralichenicidin, AmyA1 and AmyA2 both contain possible leader regions, which end with GC and GA leader cleavage sites, respectively (Fig. 6d). Notably, a small ORF (BAMY6639_RS13810) near AmyA2 in *B. amyloliquefaciens* UMAF6639 showed 58 and 51% identity with PicA2 and AmyA2. In general, Amyloliquecidin of *B. amyloliquefaciens* revealed 58–82% identity with Paralichenicidin and remained consistent genes downstream and upstream of Paralichenicidin.

The other two possible relative gene clusters studied are located in *S. pneumoniae* and *S. himastatinicus*, respectively. *S. pneumoniae* SP23-BS72 is a clinical pneumonia-associated isolate [39]. Concerning its putative Lantipeptide-encoding gene cluster, as with Paralichenicidin, a *lanR* gene (CGSSp23BS72_03608) encoding a MutR family transcriptional regulator is located upstream of the gene cluster, but no sequence identity was detected between LanR and PicR. Downstream of the gene cluster, there are two putative LanT determinants (CGSSp23BS72_ 03643: LanT1 and CGSSp23BS72_03553: LanT2) that show 39 and 43% identity with PicT1 and PicT2, respectively, and the other four hypothetical proteins show significant homology to the homologous proteins of Paralichenicidin (Fig. 6d). Moreover, the order of LanT1, LanT2 and four hypothetical proteins is also consistent with Paralichenicidin. Significantly, LanM2 has an apparent frameshift mutant (CGSSp23BS72_03633 and CGSSp23BS72_03638) and the propeptides of the two-peptide Lantipeptide are encoded by two neighboring small ORFs between LanM1 and the frameshifted-LanM2. Two propeptides (CGSSp23BS72_03623: LanA1 and CGSSp23BS72_03628: LanA2) revealed 40 and 33% identity with PicA1 and PicA2, respectively.

*S. himastatinicus* ATCC 53653 was isolated from soil according to the ability to produce Himastatin [40]. With respect to its putative Lantipeptide-encoding gene cluster, a putative LanT determinant (SSOG_RS27830: LanT1, 35% identical to PicT1) as well as two putative propeptide determinants (SSOG_RS27830 and SSOG_RS27830, 37% identical to PicA1 and 36% identical to PicA2, respectively) are located in close proximity to two putative LanM1- and LanM2-encoding genes (SSOG_RS27830 and SSOG_RS27830, 26% identical to PicM1 and 26% identical to PicM2, respectively) (Fig. 6d). The order of these genes is the same as the homologous genes of Paralichenicidin. In general, the homologous relationship between these two putative Lantipeptide-encoding gene clusters and Paralichenicidin is, therefore, less likely due to recently HGT or convergent evolution. They seem to evolve from an ancient ancestor with a long time for subsequent divergent evolution.

As a result, sequence comparisons of these propeptides were performed with Paralichenicidin to determine whether this conservation extended to the putative homologous genes. The LanA1 peptides display a high level of conservation, especially in the lanthionine and methyllanthionine bridge-forming regions (Fig. 6e). The conservation reflects the shared mode of action, specifically in binding to lipid II [41]. LanA2 displays great levels of divergence, and only shows a low degree of conservation in the N-terminal regions (Fig. 6e). The divergence of LanA2 reflects the broader role of LanA2 in membrane insertion, and the low conservation of N-terminal regions likely plays an important role in membrane insertion and pore formation [41].

Considering the possible important role of Paralichenicidin in the antimicrobial strategy, it was particularly interesting to determine whether the core peptide coding regions of LanA1 and LanA2 were undergoing selection pressure. Thus, the codeml program of PAMLX [42] was used to carry out the analysis of positive selection. Based on the LRT statistic for comparing model M1 (neutral) with model M2 (selection) and model M7 (neutral) with model M8 (selection), the core peptide coding regions of LanA1 and LanA2 were identified to be under negative selection with the ω < 1. In addition, none positively selected sites were determined. The results of positive selection indicate the LanA1 and LanA2 have been conserved during evolution.

## Discussion

In this study, we determined the antagonistic activity of *B. paralicheniformis* MDJK30 to fungi and bacteria and tested some other general characteristics as PGPR. MDJK30 showed high antagonistic activity to *F. solani* and *B. subtilis*, which indicates the potential application for controlling pathogenic fungi and gram-positive bacteria. As a member of PGPR, MDJK30 could also produce siderophores and show casein degradation activity. *B. paralicheniformis* species have long been known for their ability to produce secondary metabolites. Here, our comparative genomic analysis identified 11 gene clusters related to secondary metabolites in MDJK30, including Lichenysin, Fengycin, Bacitracin, Bacillibactin, Lantipeptide, Bacteriocin, Siderophore, Terpene, Lassopepetide, T3pks and others (unknown). As MDJK30 colonizes plant rhizospheres, it directly inhibits the growth of plant-pathogenic bacteria or fungus though production of antimicrobial peptides or depriving them of essential iron. MDJK30 is known to produce siderophores (Fig. 1d), and this capacity contributes to their fitness in iron-limited environments [1]. Lantipeptide represents a potential antimicrobial strategy against a range of pathogenic bacteria [30]. The other gene clusters might be related to the production of new antimicrobial compounds. Interestingly, the presence of a large number of mobile genetic elements and some gene clusters related to secondary metabolites in MDJK30 show high homology to those from other species, suggesting that these clusters were horizontally transferred among *B. paralicheniformis* and other species.

We then performed comparative genomics analysis based on *B. paralicheniformis* MDJK30 and 55 other previously published *Bacillus* strains. The phylogenetic analysis and population genetic structure based on core genomes showed that most strains fall predominantly into two distinct phylogenetic lineages designated lineages L (lineage *licheniformis*) and P (lineage *paralicheniformis*) and MDJK30, and the other 13 strains formed lineage P; lineage P and L form distinct and extremely tight clusters on separate clades from other strains (Fig. 3). Our phylogenetic analysis revealed the evolutionary position of MDJK30 and the explicit evolutionary relationship among *B. licheniformis* and *B. paralicheniformis*, suggesting that species-specific genome segments of *B. licheniformis* and *B. paralicheniformis* have occurred during adaptation to different niches, and co-evolution with plants, plant-pathogenic bacteria and fungus could be an important driving factor [43]. Furthermore, six mis-classification *paralicheniformis* strains were distinguished from the *licheniformis* and were also supported by the ANI result.

In addition, our comparative genomic analysis indicated that the pan-genomes of lineage P are open. Based on the COG analysis, MDJK30 contains a larger proportion of genes involved in the transcription (category K), transport and metabolism of carbohydrate and amino acid (categories G and E). The genes related to rhizosphere adaptation during PGPR-plant interactions were mainly involved in central metabolism, secretion, detoxification and stress [44]. The diversity of carbohydrate and amino acid metabolism improves genetic fitness to adapt to the specific nutrient environment in agricultural application [45]. The core genome of lineage P has a consistent functional category by COG compared with MDJK30 and indicated that despite their geographical isolation and varied associated plants, the majority of genes implicated in rhizosphere adaptation and competitiveness were highly conserved among *B. paralicheniformis* strains.

Our comparative and evolutionary analyses of the evolutionary origin and distribution of secondary metabolism gene clusters among *paralicheniformis-licheniformis* group in pan genome revealed the commonality and differences in rhizosphere adaptation between *B. paralicheniformis* and *B. licheniformis* (Fig. 5), implying that a specific niche, such as the plant-associated rhizosphere, influences HGT processes and gene loss [46, 47]. Interestingly, two non-ribosomal peptides and one specific Lantipeptide synthase gene were found in the lineage P and were absent in other lineages, including most lineage L, are those that involved in the synthesis of Bacitracin, Fengycin and Paralichenicidin. As the secondary metabolism gene clusters displayed an apparent deviation in GC content, it is likely that they were acquired through HGT events. The genetic organization of the gene clusters was closely related to the gene cluster for Fengycin and Bacitracin biosynthesis, suggesting that they have evolved from a common ancestor and/or might be interchangeable genetic elements (Fig. 6). For the Fengycin and Bacitracin clusters, we deepened the comparative genomic analysis by performing a phylogeny. The phylogenetic analysis revealed Fengycin cluster distributed in a narrow cluster of *subtilis* group, Bacitracin cluster distributed in some species of *subtilis* and *cereus* groups (Fig. 7). The phylogenetic trees of cluster and specie presented in this work, the conserved genetic organization and uncommon cluster origin, suggesting that the Fengycin and Bacitracin clusters may have been originated via an HGT event from the donor closely related to the subtilis order and the ancestor order of *cereus, coagulans* and *subtilis* groups, respectively (Fig. 7).

Cyclic lipopeptides produced by *Bacillus* strains have been reported to protect host plants from many pathogens. The representative families of these cyclic lipopeptides (Surfactin, Fengycin and Iturin) share a polypeptide ring linked to a lipid tail of various length [48, 49]. Fengycin prevents a large number of plant pathogens, especially filamentous fungi [32]. Interestingly, there is an incomplete Fengycin cluster in some non-plant-associated strains, indicating gene loss events related to the biosynthetic genes of Fengycin production. Bacitracin is a broad-spectrum gram-positive bacterial antibiotic used extensively as a feed additive that is synthesized by the non-ribosomal peptide synthase *bac* operon by several strains of *B. licheniformis* and *B. subtilis* [11, 33]. Our comparative genomic analysis clearly illustrates the distribution of the *bac* operon in *paralicheniformis-licheniformis* group, indicating that this operon is present only in the strains of lineage P (Fig. 5b). These genes are also absent from all strains of lineage L. This indicates that Bacitracin production and erythromycin resistance could be an important characteristic specific to *B. paralicheniformis*.

Paralichenicidin is a two-peptide Lantipeptide. Comparison to Lichenicidin, Paralichenicidin shows differences in genetic organization and sequence similarity. We then investigated the putative homologs of Paralichenicidin. The result showed that amyloliquecidin of *B. amyloliquefaciens* revealed 58–82% identity with Paralichenicidin and a consistent gene order of Paralichenicidin (Fig. 6d), possibly indicating that the Lantipeptide of *B. amyloliquefaciens* and *B. paralicheniformis* are evolved from a common ancestor. Such differences display the evolutionary divergence between *B. amyloliquefaciens* and *B. paralicheniformis.* There are two other possible relative gene clusters located in *S. pneumoniae* and *S. himastatinicus*, respectively. We compared the protein sequences of these propeptides of Paralichenicidin to the putative homologous proteins (Fig. 6e). The LanA1 peptides display a high degree of conservation, reflecting the shared mode of action in specifically binding to lipid II [41]. LanA2 displays a high level of divergence, and the low conservation of the N-terminal regions likely plays an important role in membrane insertion and pore formation [41]. Based on the analysis of positive selection, the *lanA1* and *lanA2* of Paralichenicidin were identified to be under negative selection with the ω < 1, and indicating they were conserved during evolution.

As a result, Fengycin production, erythromycin resistance related to Bacitracin production, and Paralichenicidin production, may therefore be useful for differentiating *B. paralicheniformis* from *B. licheniformis.* The phylogenetic analysis between cluster tree and species tree, narrow distribution, conserved genetic organization and uncommon cluster origin, suggesting that these clusters may have been originated via HGT event. These results indicate that *B. paralicheniformis* has evolved specific genomic and metabolic features and obtained gene clusters and metabolites that are distinct from *B. licheniformis*. Our data provide differences at molecular level for the production of secondary metabolites between *B. paralicheniformis* and *B. licheniformis*.

## Conclusion

*B. paralicheniformis* MDJK30 possesses several beneficial properties and has potential to enhance plant growth. The complete genome sequence of *B. paralicheniformis* MDJK30, along with the comparative genomics analysis, suggest that MDJK30 has diverse metabolic pathways and can utilize various energy sources. It can also synthesize siderophores for iron competition. Thus, MDJK30 can be developed and commercially formulated, either alone or as part of microbial consortia, for field application to control plant pathogens and promote crop growth. The explicit classification status of *B. licheniformis* and *B. paralicheniformis* was also confirmed by phylogenetic analysis, population structure analysis and ANI values. The most striking difference in the aspect of *B. paralicheniformis* is the presence of Bacitracin, Fengycin and Paralichenicidin synthetases. These synthetases are apparently not present in *B. licheniformis*. The origin and evolution of secondary metabolites clusters is another interesting topic, and results suggest that HGT events may have shaped the metabolic potential of *B. paralicheniformis*. Our study offers a new perspective regarding genomic differences between *B. paralicheniformis* and *B. licheniformis* in terms of rhizosphere adaptation.

## Methods

### Bacterial strains

*B. paralicheniformis* MDJK30 was isolated from the rhizosphere of a peony and preserved in our lab (Shandong Key Laboratory of Agricultural Microbiology, Shandong Agricultural University). The complete genome sequence of MDJK30 was sequenced and deposited at GenBank (accession number CP020352) [14]. Our collection for constructing core genome phylogeny included 9 *B. paralicheniformis* strains, 46 *B. licheniformis* strains and 1 *B. sonorensis* strains (Fig. 3). Twenty other *Bacillus* genomes were used in constructing phylogeny of Species, Fengycin, and Bacitracin (Fig. 7). All the genomes of other strains were downloaded from the NCBI GenBank database. Bacterial strains used in this study are presented in Additional file 4: Table S2.

### Analysis of the antagonistic activity

A dual culture assay [50] was performed to analyze the antagonistic activity of *Bacillus paralicheniformis* MDJK30. *Fusarium solani* from the root rhizosphere of peony was used to test the antifungal activity of MDJK30. Hyphal plugs of *F. solani* were cut from a new cultivation and placed in the center of a PDA plate to incubate for 1 day at 28 °C. Then, a single clone of MDJK30 was inoculated onto one side of the plug at a distance of 2 cm to incubate for another 3 days at 28 °C. The model organisms *E. coli* DH5α and *B. subtilis* 168 were used for antibacterial tests of MDJK30. The precultured *E. coli* or *B. subtilis* were then cultured in 5 mL LB liquid medium for 10 h at 37 °C. Then, 1 mL of the culture was mixed with 20 mL LB semisolid medium, and a single colony of MDJK30 was then placed on the center of the cooled medium to incubate for 1 day at 37 °C. The inhibition zones were measured.

### Qualitative analysis of siderophores

Single clones of *B. paralicheniformis* MDJK30 were cultivated on LB plates overnight at 37 °C. The bacterial lawn was inoculated on a CAS-agar plate for qualitative analysis of siderophores, as reported [51].

### Casein degradation assay

Casein degradation of *B. paralicheniformis* MDJK30 was tested on the casein medium, which contained 1% (*w*/*v*) casein, 0.3% (w/v) beef extracts, 0.5% (w/v) NaCl, 0.2% (w/v) K_2_HPO_4_, 2% (w/v) agar, and 0.005% (w/v) bromothymol blue, pH 7.3–7.5. Single clones of MDJK30 were inoculated on the casein medium and cultivated for 2 days at 37 °C to observe the transparent zones that indicated the capacity for degrading casein.

### Identification of gene orthologous group

Orthologous groups were delimited using OrthoFinder [52], in which all protein sequences were compared using a BLASTP all-against-all search with an E-value cutoff of 1e-3. The single-copy core gene, pan genome and core genome set were extracted from the OrthoFinder output. Nucleotide sequences of the single-copy core genes were extracted according to protein ID in the OrthoFinder output.

### Phylogenetic analysis

The phylogenetic relationship between the *Bacillus* strains was predicted by analyzing the set of SNPs present in all single-copy core genes across genomes. The SNPs were integrated according to the arrangement of the single-copy genes on MDJK30 genome. Homologous recombination caused by HGT occurs in bacterial populations and can confound phylogenetic analysis. Extracting the set of SNPs, we identified and removed putative regions of recombination, using the tool CloneFrameML [53]. Phylogenetic trees were constructed using two methods: Maximum likelihood (ML) and Neighbor Joining (NJ). The nucleotide sequence of genes used in the phylogenetic tree were aligned using MAFFT [54]. ML tree was constructed using PhyML [19], with the GTR (General Time Reversible) model of nucleotide substitution, c-distributed rates among site. NJ tree was computed by applying a Poisson model available with 100 bootstrap replicates and uniform rates in MEGA7 [55]. MEGA7 and FigTree 1.4.3 (http://tree.bio.ed.ac.uk/software/figtree/) were employed to show the trees. The choice of models was based on previous studies [56, 57].

### Population structure analysis

The population genetic structure of 56 genomes was investigated using the software BAPS (version 6) [20] and STRUCTURE (version 2.3.4) [21] based on SNPs identified from the alignment of 528 single-copy core genes shared by the 56 genomes and integrated according to the arrangement of the genes on MDJK30 genome. BAPS assigns strains to inferred populations (K), representing the best fit for the observed genetic variation. We varied K from 2 to 10 and ran the experiment three times to confirm the clustering results. We ran the program STRUCTURE at values of k (the number of subpopulations 2–10) and Rep (repeats 5). STRUCTURE assumed k = 6 subpopulations and correlated allele frequencies, linkage model based on maker distances in base pairs, 10,000-iteration burnin and 10,000 iterations of sampling.

### Average nucleotide identity (ANI) analysis

Average nucleotide identity (ANI), a method that can be applied to delineate species, was calculated to determine diversity at the genomic level [58]. JSpecies1.2.1 was used to analyze these genome sets for ANI and tetramer usage pattern, using the default parameters [58].

### Functional category of genes

We analyzed the functional category of the genes of MDJK30 and core genome of lineage P using Cluster of Orthologous Group (COG) assignment [26]. The functional annotation of proteins was performed by alignment against the COG database of NCBI using amino acid sequences.

### The secondary metabolisms analysis

The gene cluster related to secondary metabolism was identified and the putative structure was deduced using antiSMASH on the default parameters [16] (http://antismash.secondarymetabolites.org). The results obtained from genomic sequence correlated with NRPS pathway consisted of detailed functional domain annotation, predicted core structure, and the levels of genomic identity to known homologous gene clusters catalogued in the Minimum Information on Biosynthetic Gene Cluster (MIBiG).

### Comparative genomics

Genomic features were visualized using DNAplotter [59]. The circular maps were generated using BLAST Ring Image Generator (BRIG) software [60]. The PHAge search tool (PHAST) was utilized to find the prophages [61]. Genomic islands were predicted using IslandViewer [62]. Insertion sequences were predicted using the IS Finder database [63]. The clustered regularly interspaced short palindromic repeats (CRISPRs) were predicted with the CRISPR recognition tool (CRT) [64].

To examine the evolutionary origin and distribution of the secondary metabolism gene cluster, we located and screened the biosynthetic and transport-related genes and regions of gene clusters using the LS-BSR tool [65].

### Genome mining of LanM-like enzymes

To identify LanM-like enzymes of Paralichenicidin, PSI-BLAST searches of the NCBI non-redundant protein database were performed using the LanM protein sequences as the query. Hits were selected with E-values lower than 1e-6.

### Analysis for positive selection

MEGA [55] was used to construct maximum likelihood phylogenetic trees with the GTR model of nucleotide substitution for the core peptide coding region of LanA1 and LanA2. The resulting ML trees were applied to subsequent selection analysis. Positive selection was assessed in the program codeml in the PAMLX [42]. Positive selection in coding regions can be estimated by calculating the ratio of the nonsynonymous substitution rate to the synonymous substitution rate (*dN*/*dS*, represented by the omerga parameter in codeml.). The likelihood ratio test (LRT) were then carried out to infer the occurrence of core peptide coding region under positive selection pressure through two different comparisons of model M1 (neutral) with model M2 (selection) and model M7 (neutral) with model M8 (selection). *P* values were determined from the LRT scores calculated by the module *Chi-square* of the PAMLX [42].

## Additional files

Additional file 1: **Table S1.** The features of prophage, genomic islands, secondary metabolite clusters and CRISPR in MDJK30 genome. (XLSX 14 kb)

Additional file 2: **Figure S1.** A: Biosynthetic gene clusters and predicted structures for NRPS in MDJK30. B: Other biosynthetic gene clusters for secondary metabolism in MDJK30. Eleven gene clusters for secondary metabolism were predicted using antiSMASH, designated Lichenysin, Fengycin, Bacitracin, Bacillibactin, Lantipeptide, Bacteriocin, Siderophore, Terpene, Lassopepetide, T3pks and Other (unknown). (PDF 1541 kb)

Additional file 3: **Figure S2.** Comparative analysis of biosynthetic gene clusters for secondary metabolism from MDJK30 and other strains. Different genes are in different colors and genes with the same color are homologous to each other. (PDF 1320 kb)

Additional file 4: **Table S2.** Genetic characteristics of strains in the current research. (XLSX 15 kb)

Additional file 5: **Table S3.** List of 528 single-copy core genes shared by 56 *Bacillus* strains. (XLSX 37 kb)

Additional file 6: **Table S4.** Table S4. Average Nucleotide Identity (ANI) (%) based on whole-genome alignments. ANI values of lineage L are in blue and lineage P are in purple. (XLSX 62 kb).

Additional file 7: **Table S5.** List of 1718 single-copy core genes shared by 14 lineage P strains. (XLSX 97 kb).

Additional file 8: **Figure S3.** A: Neighbor-joining phylogenetic tree of 47 *Bacillus* genome. B: NJ tree of the 14 lineage P strains. (PDF 635 kb)

Additional file 9: **Figure S4.** Comparison of GC content between gene clusters of Fengycin, Bacitracin and Paralichenicidin and coding regions of genome. (PDF 895 kb)

## Abbreviations

ANI: Average nucleotide identity
*B. licheniformis*: *Bacillus licheniformis*
*B. paralicheniformis*: *Bacillus paralicheniformis*
*B. sonorensis*: *Bacillus sonorensis*
*B. subtilis*: *Bacillus subtilis*
BAPS: Bayesian Analysis of Population Structure
BSR: Blast score ratio
CAS: chrome azurol S
CDS: Coding DNA sequence
COG: Cluster of orthologous group
CRISPR: clustered regularly interspaced short palindromic repeats
*E. coli*: *Escherichia coli*
*F.solani*: *Fusarium solani*
GRT: General Time Reversible
HGT: horizontal gene transfer
IS: Insertion sequence
LB: Luria-Bertani
lineage L: lineage *licheniformis*
lineage P: lineage *paralicheniformis*
NRPS: Nonribosomal peptide synthetases
PDA: Potato Dextrose Agar
PGPR: Plant growth-promting rhizobacteria
PKS: Polyketide synthases
