# Supplementary material for: Comparative genomic analysis of Bacillus paralicheniformis MDJK30 with its closely related species reveals an evolutionary relationship between B. paralicheniformis and B. licheniformis
Source: BMC Genomics. 2019 Apr 11;20:283. doi: 10.1186/s12864-019-5646-9 (PMC6458615; doi:10.1186/s12864-019-5646-9)
Supplement: Supplementary file 2 — Figure S1. A: Biosynthetic gene clusters and predicted structures for NRPS in MDJK30. B: Other biosynthetic gene clusters for secondary metabolism in MDJK30. Eleven gene clusters for secondary metabolism were predicted using antiSMASH, designated Lichenysin, Fengycin, Bacitracin, Bacillibactin, Lantipeptide, Bacteriocin, Siderophore, Terpene, Lassopepetide, T3pks and Other (unknown). (PDF 1541 kb) [file 12864_2019_5646_MOESM2_ESM.pdf]

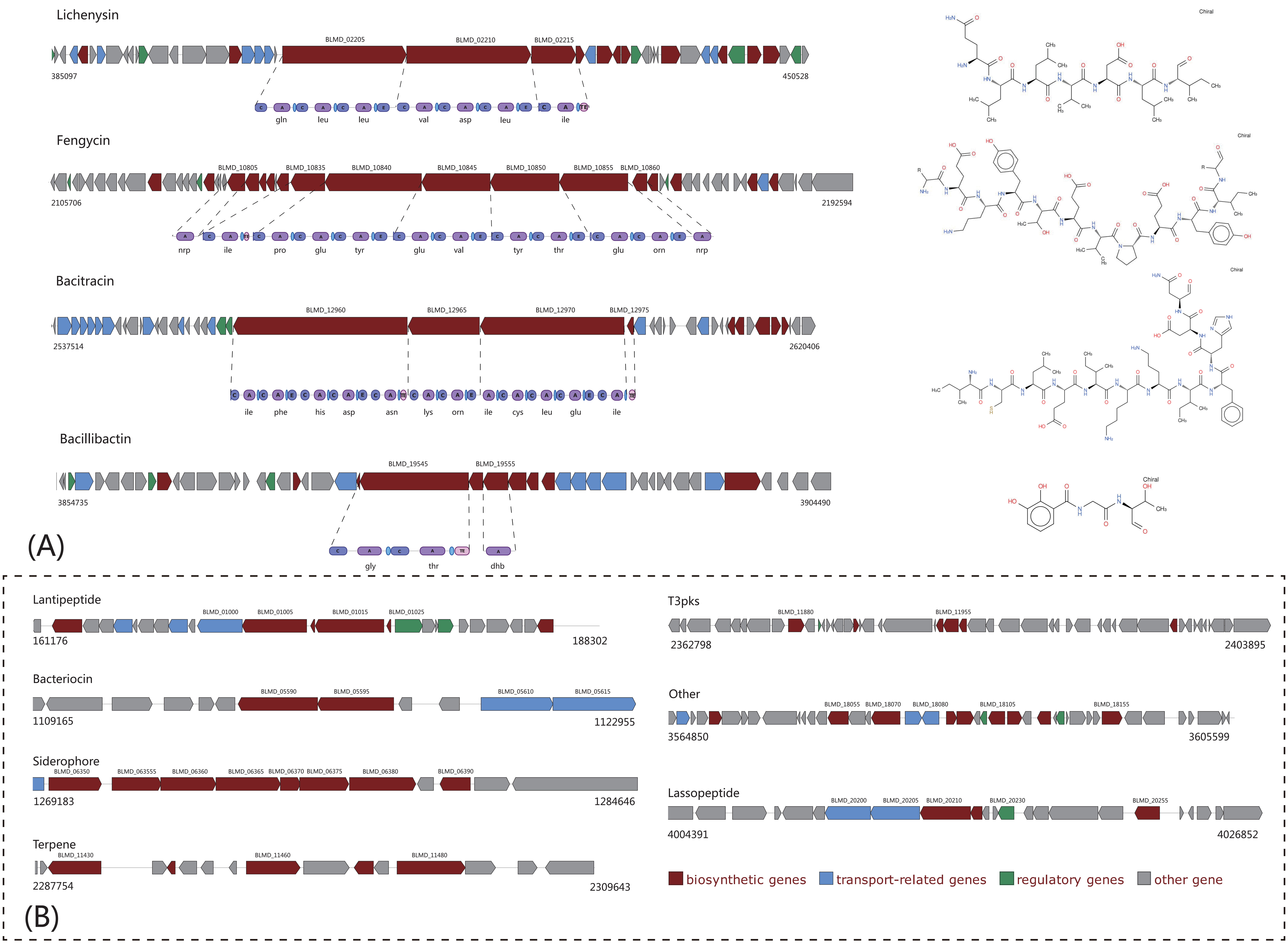

Lichenysin
BLMD_02205
BLMD_02210
BLMD_02215
385097
450528
gln
leu
leu
val
asp
leu
ile
Chiral
Fengycin
BLMD_10805
BLMD_10835
BLMD_10840
BLMD_10845
BLMD_10850
BLMD_10855
BLMD_10860
2105706
2192594
nrp
ile
pro
glu
tyr
glu
val
tyr
thr
glu
orn
nrp
Chiral
Bacitracin
BLMD_12960
BLMD_12965
BLMD_12970
BLMD_12975
2537514
2620406
ile
phe
his
asp
asn
lys
orn
ile
cys
leu
glu
ile
Chiral
Bacillibactin
BLMD_19545
BLMD_19555
3854735
3904490
gly
thr
dhb
Chiral
(A)
Lantipeptide
BLMD_01000
BLMD_01005
BLMD_01015
BLMD_01025
161176
188302
Bacteriocin
BLMD_05590
BLMD_05595
BLMD_05610
BLMD_05615
1109165
1122955
Siderophore
BLMD_06350
BLMD_063555
BLMD_06360
BLMD_06365
BLMD_06370
BLMD_06375
BLMD_06380
BLMD_06390
1269183
1284646
Terpene
BLMD_11430
BLMD_11460
BLMD_11480
2287754
2309643
T3pks
BLMD_11880
BLMD_11955
2362798
2403895
Other
BLMD_18055
BLMD_18070
BLMD_18080
BLMD_18105
BLMD_18155
3564850
3605599
Lassopeptide
BLMD_20200
BLMD_20205
BLMD_20210
BLMD_20230
BLMD_20255
4004391
4026852
biosynthetic genes
transport-related genes
regulatory genes
other gene
(B)
