# Supplementary material for: Comparative genomic analysis of Bacillus paralicheniformis MDJK30 with its closely related species reveals an evolutionary relationship between B. paralicheniformis and B. licheniformis
Source: BMC Genomics. 2019 Apr 11;20:283. doi: 10.1186/s12864-019-5646-9 (PMC6458615; doi:10.1186/s12864-019-5646-9)
Supplement: Supplementary file 3 — Figure S2. Comparative analysis of biosynthetic gene clusters for secondary metabolism from MDJK30 and other strains. Different genes are in different colors and genes with the same color are homologous to each other. (PDF 1320 kb) [file 12864_2019_5646_MOESM3_ESM.pdf]

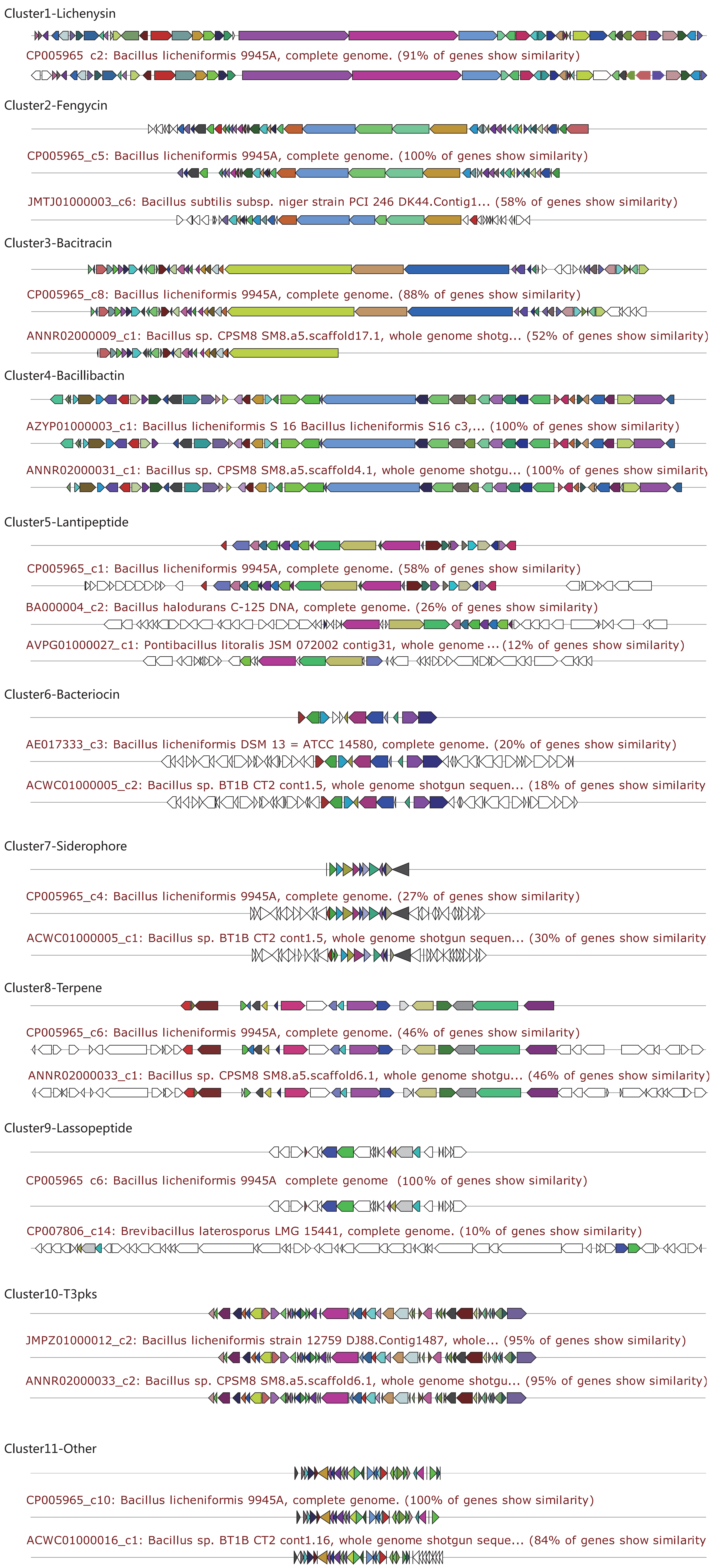

Cluster1-Lichenysin
CP005965 c2: Bacillus licheniformis 9945A, complete genome. (91% of genes show similarity)
Cluster2-Fengycin
CP005965_c5: Bacillus licheniformis 9945A, complete genome. (100% of genes show similarity)
JMTJ01000003_c6: Bacillus subtilis subsp. niger strain PCI 246 DK44.Contig1... (58% of genes show similarity)
Cluster3-Bacitracin
CP005965_c8: Bacillus licheniformis 9945A, complete genome. (88% of genes show similarity)
ANNR02000009_c1: Bacillus sp. CPSM8 SM8.a5.scaffold17.1, whole genome shotg... (52% of genes show similarity)
Cluster4-Bacillibactin
AZYP01000003_c1: Bacillus licheniformis S 16 Bacillus licheniformis S16 c3,... (100% of genes show similarity)
ANNR02000031_c1: Bacillus sp. CPSM8 SM8.a5.scaffold4.1, whole genome shotgu... (100% of genes show similarity
Cluster5-Lantipeptide
CP005965_c1: Bacillus licheniformis 9945A, complete genome. (58% of genes show similarity)
BA000004_c2: Bacillus halodurans C-125 DNA, complete genome. (26% of genes show similarity)
AVPG01000027_c1: Pontibacillus litoralis JSM 072002 contig31, whole genome ... (12% of genes show similarity)
Cluster6-Bacteriocin
AE017333_c3: Bacillus licheniformis DSM 13 = ATCC 14580, complete genome. (20% of genes show similarity)
ACWC01000005_c2: Bacillus sp. BT1B CT2 cont1.5, whole genome shotgun sequen... (18% of genes show similarity
Cluster7-Siderophore
CP005965_c4: Bacillus licheniformis 9945A, complete genome. (27% of genes show similarity)
ACWC01000005_c1: Bacillus sp. BT1B CT2 cont1.5, whole genome shotgun sequen... (30% of genes show similarity
Cluster8-Terpene
CP005965_c6: Bacillus licheniformis 9945A, complete genome. (46% of genes show similarity)
ANNR02000033_c1: Bacillus sp. CPSM8 SM8.a5.scaffold6.1, whole genome shotgu... (46% of genes show similarity)
Cluster9-Lassopeptide
CP005965 c6: Bacillus licheniformis 9945A complete genome (100% of genes show similarity)
CP007806_c14: Brevibacillus laterosporus LMG 15441, complete genome. (10% of genes show similarity)
Cluster10-T3pks
JMPZ01000012_c2: Bacillus licheniformis strain 12759 DJ88.Contig1487, whole... (95% of genes show similarity)
ANNR02000033_c2: Bacillus sp. CPSM8 SM8.a5.scaffold6.1, whole genome shotgu... (95% of genes show similarity)
Cluster11-Other
CP005965_c10: Bacillus licheniformis 9945A, complete genome. (100% of genes show similarity)
ACWC01000016_c1: Bacillus sp. BT1B CT2 cont1.16, whole genome shotgun seque... (84% of genes show similarity
