# Supplementary figures and images for: Comparative genomic analysis of Bacillus paralicheniformis MDJK30 with its closely related species reveals an evolutionary relationship between B. paralicheniformis and B. licheniformis
Source: BMC Genomics. 2019 Apr 11;20:283. doi: 10.1186/s12864-019-5646-9 (PMC6458615; doi:10.1186/s12864-019-5646-9)

(A)

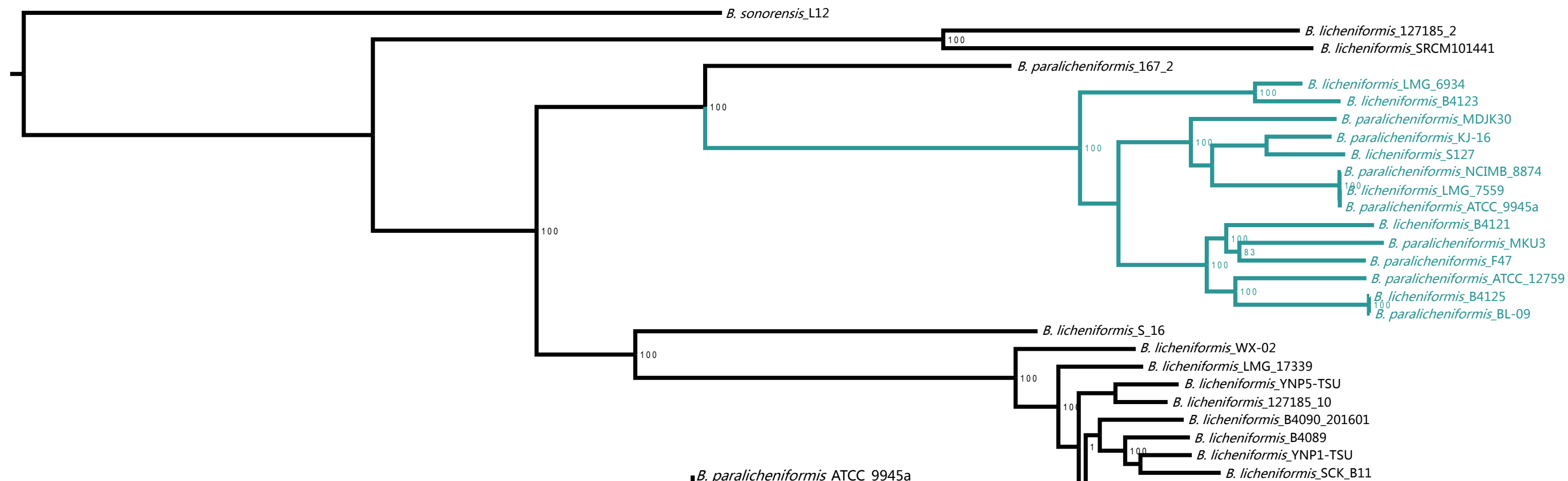

(B)

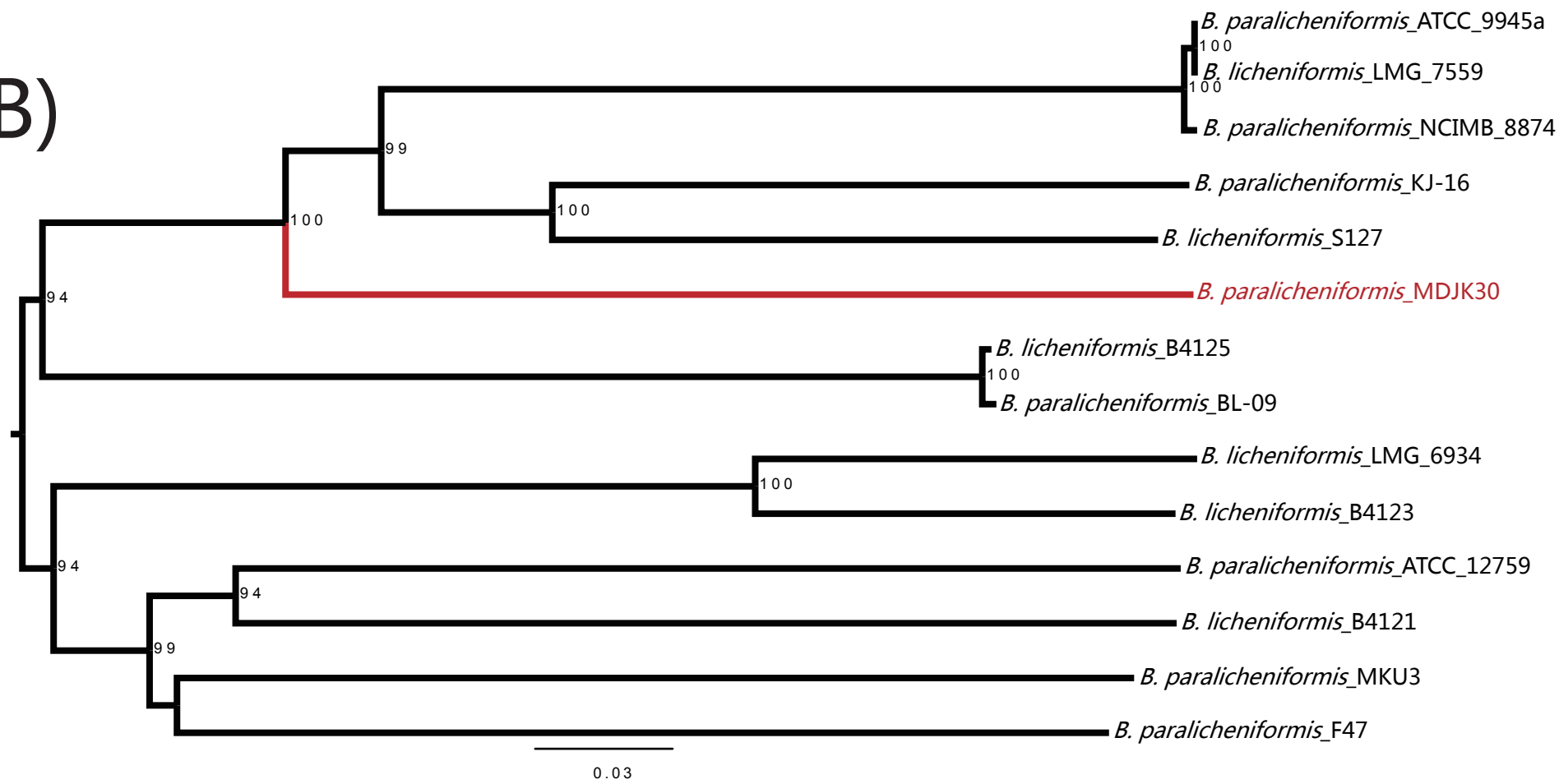

Supplement: Supplementary file 8 — Figure S3. A: Neighbor-joining phylogenetic tree of 47 Bacillus genome. B: NJ tree of the 14 lineage P strains. (PDF 635 kb) [file 12864_2019_5646_MOESM8_ESM.pdf]

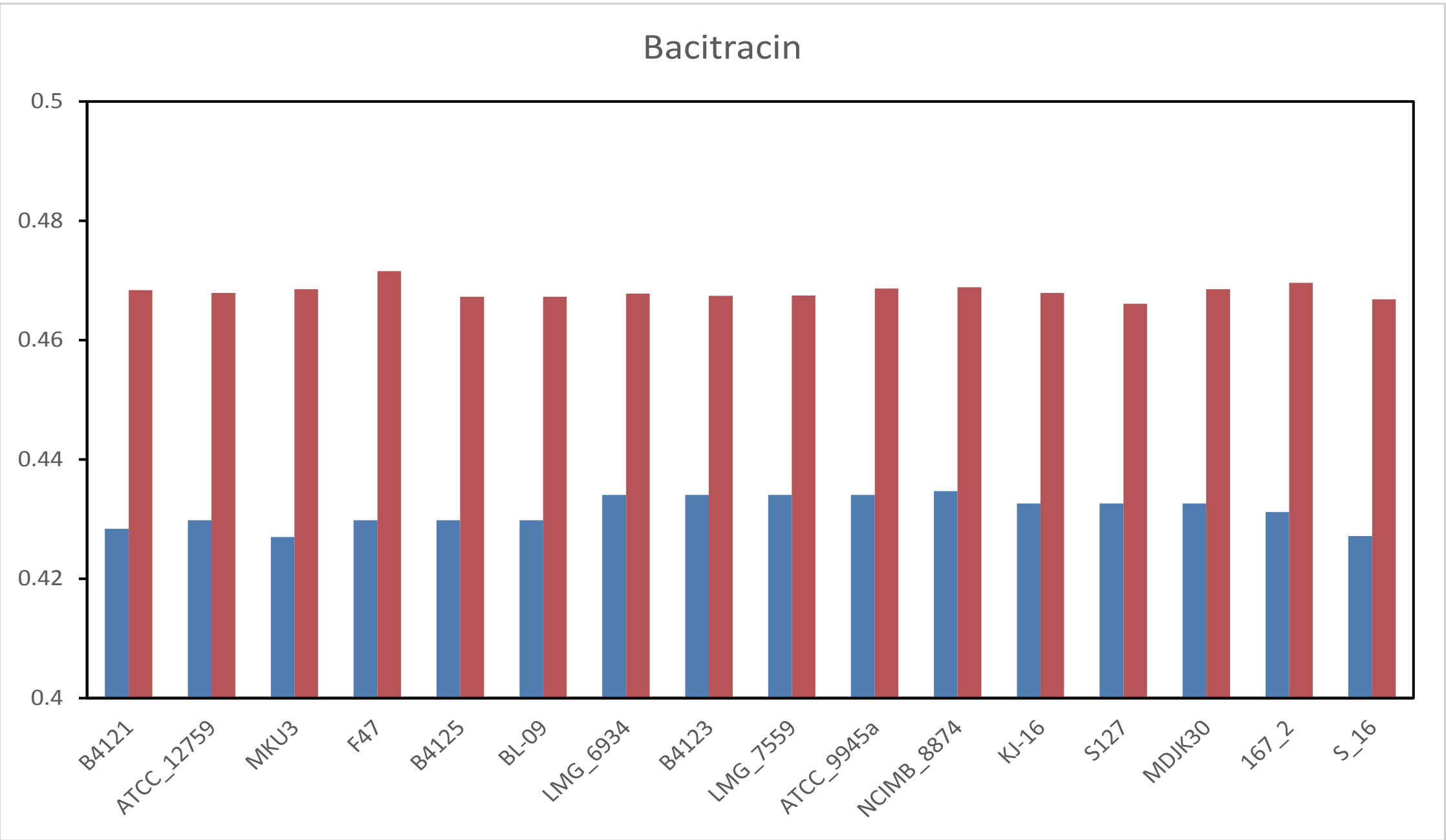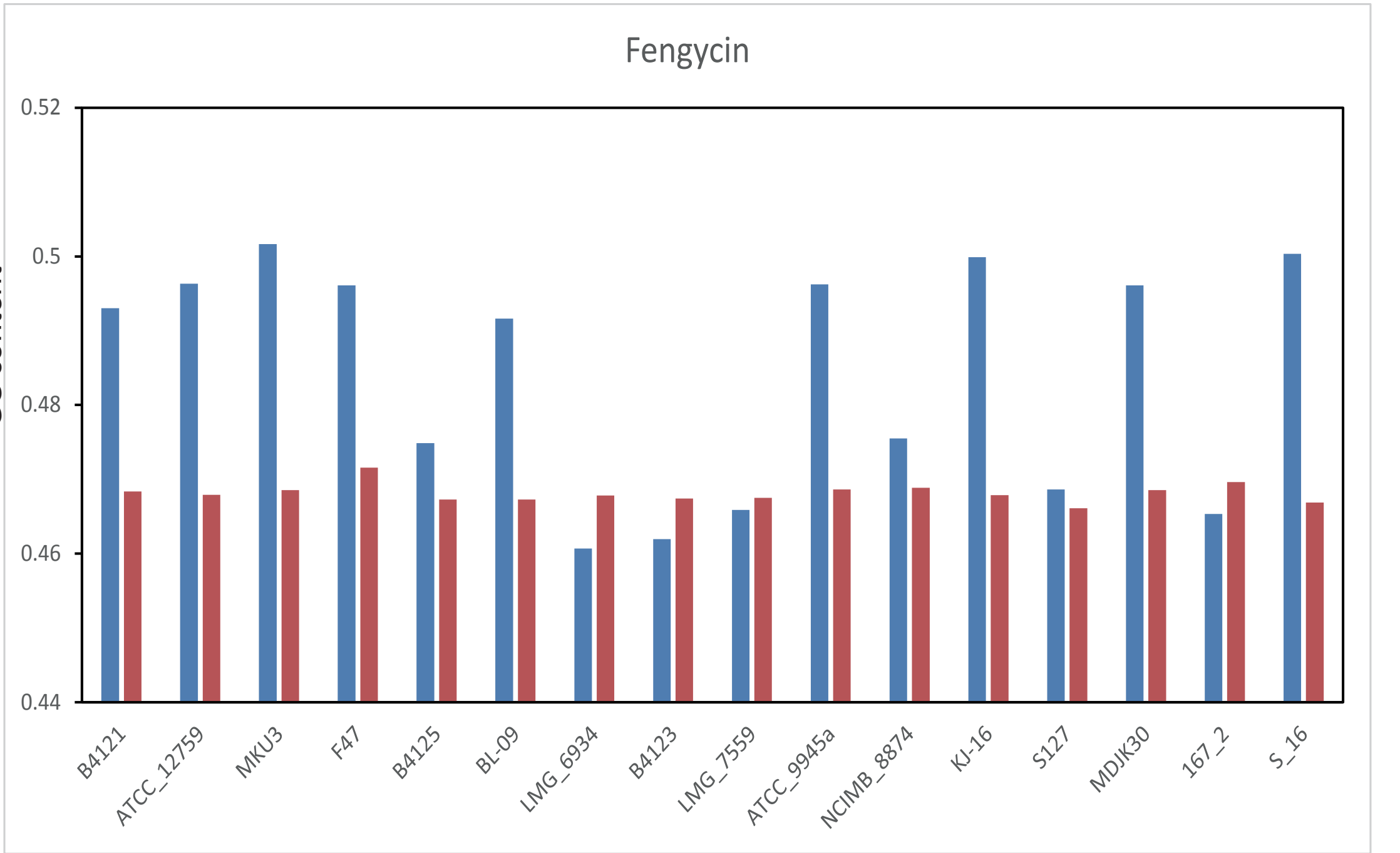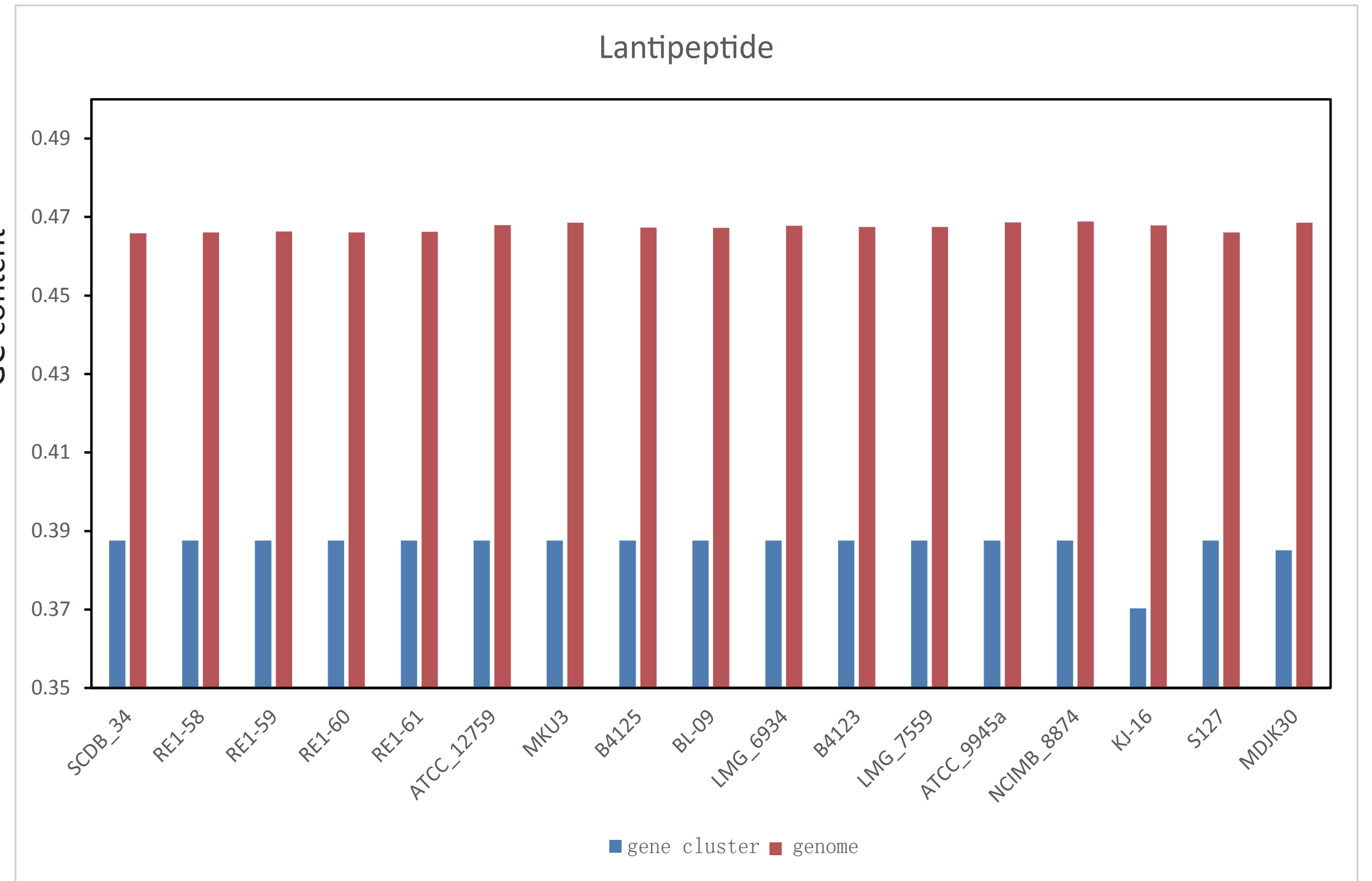

Supplement: Supplementary file 9 — Figure S4. Comparison of GC content between gene clusters of Fengycin, Bacitracin and Paralichenicidin and coding regions of genome. (PDF 895 kb) [file 12864_2019_5646_MOESM9_ESM.pdf]
